# Efficacy of *N*-Acetylcysteine in Polycystic Ovary Syndrome: Systematic Review and Meta-Analysis

**DOI:** 10.3390/nu17020284

**Published:** 2025-01-14

**Authors:** Isabel Viña, Juan R. Viña, Macarena Carranza, Gonzalo Mariscal

**Affiliations:** 1 IVB Wellness Lab, C/Colón 12, 46004 Valencia, Spain; 2 Departamento de Bioquímica y Biología Molecular, Facultad de Medicina, Instituto INCLIVA, Universitat de València, 46010 Valencia, Spain; 3 School of Medicine, Valencia Catholic University, C/Quevedo 2, 46001 Valencia, Spain; gonzalo.mariscal@mail.ucv.es

**Keywords:** polycystic ovary syndrome, *N*-acetylcysteine, fertility, metformin, PCOS, NAC

## Abstract

Background: Polycystic ovary syndrome (PCOS) is a common endocrine disorder that affects women of reproductive age and requires better treatment. *N*-acetylcysteine (NAC) is known to be beneficial under such conditions owing to its antioxidant potential and insulin-sensitizing properties. The effect of NAC on the reproductive outcomes of PCOS patients was examined in this meta-analysis. Methods: In accordance with PRISMA standards, this meta-analysis included studies that compared *N*-acetylcysteine, metformin, clomiphene citrate, and a placebo in patients with POCS. The main indicators were follicular growth, endometrial thickness, and hormone level. The risk of bias was evaluated using the Cochrane ROB2 tool. Results: Twenty-two studies (*n* = 2515) were included. NAC was associated with a statistically significant increase in progesterone (SMD 0.95, 95% CI: 0.13–1.77, *p* = 0.02) and endometrial thickness (SMD 0.58, 95% CI: 0.10–1.06, *p* = 0.02) compared to the placebo and other drugs (SMD 0.71, 95% CI: 0.48–0.94, *p* < 0.0001). LH levels were significantly increased by NAC compared to metformin (SMD 0.67, 95% CI: 0.23–1.12, *p* = 0.003). However, no significant differences were observed in the estradiol, SHBG, or FSH levels. Conclusions: NAC had a major effect on progesterone, endometrial thickness, and LH levels in women with PCOS. Therefore, it may be a potential treatment option.

## 1. Introduction

Polycystic ovary syndrome (PCOS) is the most frequently diagnosed endocrine disorder in women of reproductive age and is characterized by a complex interplay of hormonal, metabolic, and reproductive dysfunctions [1]. The pathophysiology of PCOS is driven by an increase in the frequency of luteinizing hormone (LH) pulses at the expense of follicle-stimulating hormone (FSH) secretion [2]. Hormonal imbalance disrupts ovarian function and impedes follicular maturation and ovulation [3]. Elevated LH levels stimulate androgen production by theca interna and stromal ovarian cells, resulting in hyperandrogenemia [4]. Additionally, this increase in androgens is exacerbated by a reduction in the activity of ovarian aromatase, the enzyme responsible for converting androgens into estrogens [5]. This dual mechanism amplifies androgen levels and perpetuates the symptoms of PCOS.

Hyperandrogenemia associated with PCOS is characterized by insulin resistance and is present in a significant proportion of PCOS patients [6]. Insulin acts synergistically with LH at the ovarian level to further increase androgen production, while simultaneously inhibiting follicular growth. Additionally, insulin suppresses the production of sex hormone-binding globulin (SHBG), increasing the bioavailability of androgens in circulation [7]. This combination of hormonal and metabolic abnormalities manifests clinically as a spectrum of symptoms, including irregular menstruation, infertility, increased cardiovascular risk, and metabolic syndrome [8].

Current management strategies for PCOS focus on symptom relief and address specific metabolic or reproductive concerns [9]. Treatment with metformin for insulin resistance and clomiphene citrate for ovulation induction remains the cornerstone of therapy [10]. However, other alternatives are also being explored, including anastrozole, an aromatase inhibitor that reduces estrogen synthesis, increases FSH levels, and promotes follicular development and ovulation [11]. Although anastrozole has shown promise, these interventions are not universally effective, resulting in many women with persistent symptoms and suboptimal reproductive outcomes.

Therefore, *N*-acetylcysteine (NAC) has emerged as a potential therapeutic agent [12]. Owing to its antioxidant, anti-inflammatory, and insulin-sensitizing properties, NAC functions as a precursor of glutathione, which is the most potent endogenous antioxidant [13]. Preliminary evidence suggests that NAC may reduce total testosterone levels, enhance FSH secretion, and improve metabolic and reproductive outcomes in PCOS [14]. However, existing studies are often limited by methodological heterogeneity and publication bias, making it challenging to draw definitive conclusions regarding efficacy.

A recent meta-analysis (2023) [15] highlighted the potential benefits of NAC, including reduced testosterone levels and increased FSH levels, while also identifying gaps in the literature concerning other reproductive outcomes such as endometrial thickness and estradiol levels. This underscores the need for further systematic evaluation to assess the role of NAC relative to established treatments, such as metformin, clomiphene citrate, and anastrozole.

This review aimed to address these gaps by systematically analyzing the available evidence on the effectiveness of NAC in treating PCOS. Specifically, it compared clinically significant endpoints, including hormonal regulation, ovulation rates, and metabolic improvements, across interventions involving NAC, a placebo, metformin, clomiphene citrate, and anastrozole. In doing so, we sought to clarify whether NAC can offer a meaningful therapeutic advantage in managing this multifaceted syndrome.

## 2. Material and Methods

### 2.1. Eligibility Criteria

The current systematic review and meta-analysis were conducted according to the Preferred Reporting Items for Systematic Reviews and Meta-Analyses (PRISMA) [16]. The inclusion criterion was the PICO formula. The population was female patients with PCOS. Interventions included NAC or a combination of NAC. The comparator was another drug for the treatment of PCOS, such as metformin, clomiphene citrate (CC), a placebo, or other drugs (l-carnitine). The outcomes were efficacy outcomes that reflected fertility. A comparative study design was used. 

Our exclusion criteria were any secondary or primary non-comparative studies. In addition, we excluded case reports or descriptive studies and Lab, Tissue, Cell, or animal studies. Papers that did not meet the inclusion criteria were also excluded. We restricted the included studies to only English articles because of the lack of feasibility of translating and using data from non-English articles correctly, which may have affected the results. Therefore, we excluded non-comparables to maintain consistency in study design, interventions, and outcome measures for meaningful comparisons. Reviews were excluded because they provided a summary of the existing literature rather than primary data. Studies with incomplete or missing data were excluded to maintain the integrity and reliability of the analyses. Finally, studies that did not share variables in at least two of the included studies were excluded to enable meaningful pooling of data and facilitate robust statistical analysis.

### 2.2. Information Sources and Search Methods for Identification of Studies

A systematic comprehensive search was conducted with no restriction to the start date until November 2024 using multiple databases, including PubMed, Scopus, Web of Science, and Cochrane. In the search strategy, we used the following mesh terms; [(“*N*-acetylcysteine” OR “*N*-acetyl L-cysteine”) AND (“fertility” OR “reproductive” OR “polycystic ovary syndrome”)]. In addition to the online database search, a manual search was employed to find relevant studies. In addition, the reference list of the included studies was revised to avoid missing relevant studies. Two independent reviewers screened the titles and abstracts for eligibility criteria, and a third opinion was considered in the case of conflict between the two reviewers. In addition, two reviewers independently conducted the assessment process of the full-text screening, and in cases of disagreement, a third reviewer was consulted to reach consensus.

### 2.3. Data Extraction and Data Items

Online Google Sheets were used to present the extraction sheet and summary table. The baseline characteristics included the name of the first author, year of publication, study design, study location, sample size, mean age, body mass index (BMI), and type of intervention and comparator. The main outcomes were the number of follicles, endometrial thickness, and hormone levels, including total testosterone (TT), progesterone, serum luteinizing hormone (LH), serum follicle-stimulating hormone (FSH), SHBG, and estradiol (E2). Outcome measurements at specified time points were determined, and disagreements were resolved by mutual consensus and, when required, with the help of a third person during the extraction.

### 2.4. Assessment of Risk of Bias in Included Studies

Quality assessment of the included studies was conducted using the Cochrane Collaboration Risk of Bias Tool (ROB2 tool). The following six domains were evaluated: random sequence generation, allocation concealment, blinding of participants and personnel, blinding of outcome assessments, incomplete outcome data, and selective reporting. The risk of bias was represented by the assessment of the risk of bias (ROB) graph using Review Manger 5.4 software.

### 2.5. Assessment of Results

We used Review Manager 5.4 software to conduct the meta-analysis. Means and standard deviations were used as continuous variables. Odds ratios and risk ratios were used as dichotomous variables. A 95% confidence interval was used to ensure precision of the results. Heterogeneity was assessed using Cochran’s Q test (Chi2) and *I*^2^ tests. *I*^2^ values of >25%, 50%, and 75% represented low, moderate, and high heterogeneity, respectively. We used a fixed model with no significant heterogeneity and a random model with high heterogeneity. The standardized mean difference was used to limit the heterogeneity between measurement units and combinations of different drugs. The results were considered significant when the *p*-value was less than 0.05. To obtain any data from the figures, we used WebPlotDigitizer version 4.5 to extract them. Missing data were handled according to the guidelines provided in the Cochrane Handbook [17].

### 2.6. Risk of Bias Across the Studies

Publication bias assessment was performed by the Review Manger 5.4 Software. We used funnel plots to visualize the publication bias.

### 2.7. Additional Analyses

Subgroup analyses were performed considering the different types of drugs and their combinations to evaluate the efficacy of NAC compared to the other drugs. 

Sensitivity analyses were conducted across all variables by excluding the study with the largest weight to determine whether there was any significant change in the direction of the results. This analysis was applied to all groups or subgroups containing more than two studies.

Publication bias was assessed by visual inspection of funnel plots generated by Review Manager 5.4 software when the number of included studies was more than ten studies.

Additionally, the Grading of Recommendations Assessment, Development, and Evaluation (GRADE) assessment was conducted using the GRADEpro tool. The GRADE approach involves evaluating the quality of evidence and strength of recommendations, considering factors such as the risk of bias, heterogeneity, precision of results, and consistency across studies [18].

## 3. Result

### 3.1. Study Selection

**Figure 1:**
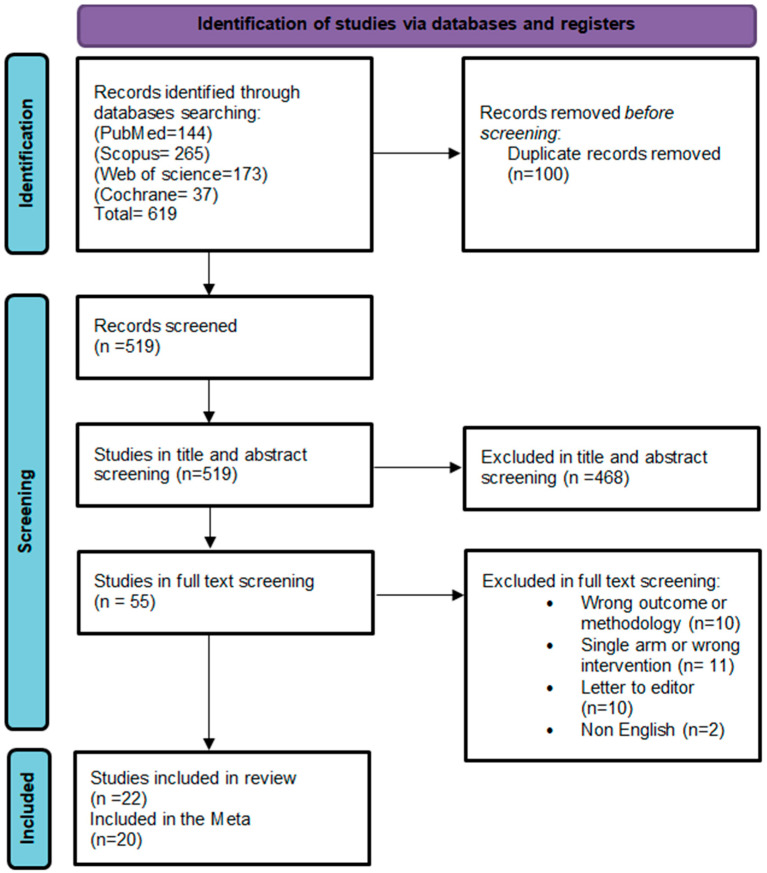
Flow diagram depicting the study selection process (Preferred Reporting Items for Systematic Reviews and Meta-Analyses) created by Microsoft word [16].

The initial electronic search across the four databases yielded 619 studies. After removing duplicates through title and abstract screening, 55 studies remained for the full-text evaluation. Of these, only 22 studies met our inclusion criteria, and 20 studies were included in the meta-analysis. No additional studies were identified during the manual search (Figure 1) [13,19,20,21,22,23,24,25,26,27,28,29,30,31,32,33,34,35,36,37,38,39].

### 3.2. Study Characteristics

Table 1 presents the baseline characteristics of the included studies. A total of 22 studies [13,19,20,21,22,23,24,25,26,27,28,29,30,31,32,33,34,35,36,37,38,39] involving 2515 patients were reviewed. The mean numbers of males and females, as well as the average age, were not consistently reported across all studies. The locations of the studies were diverse, but most (ten) were from Egypt, followed by six from Iran, three from Turkey, and one each from India, the UK, and Italy. Intervention participants were NAC alone or combined with CC, letrozole, or metformin versus the respective control groups or placebo. Study sample sizes varied between 15 and 260 participants with mean ages from 22.6 to 30.3 years and BMI ranging from 23.0 to 33.1 kg/m^2^. These studies investigated different potential treatment approaches for PCOS and fertility-related disorders using a variety of subjects and trial designs to guide translation into the clinical setting.

### 3.3. Risk of Bias Assessment

**Figure 2:**
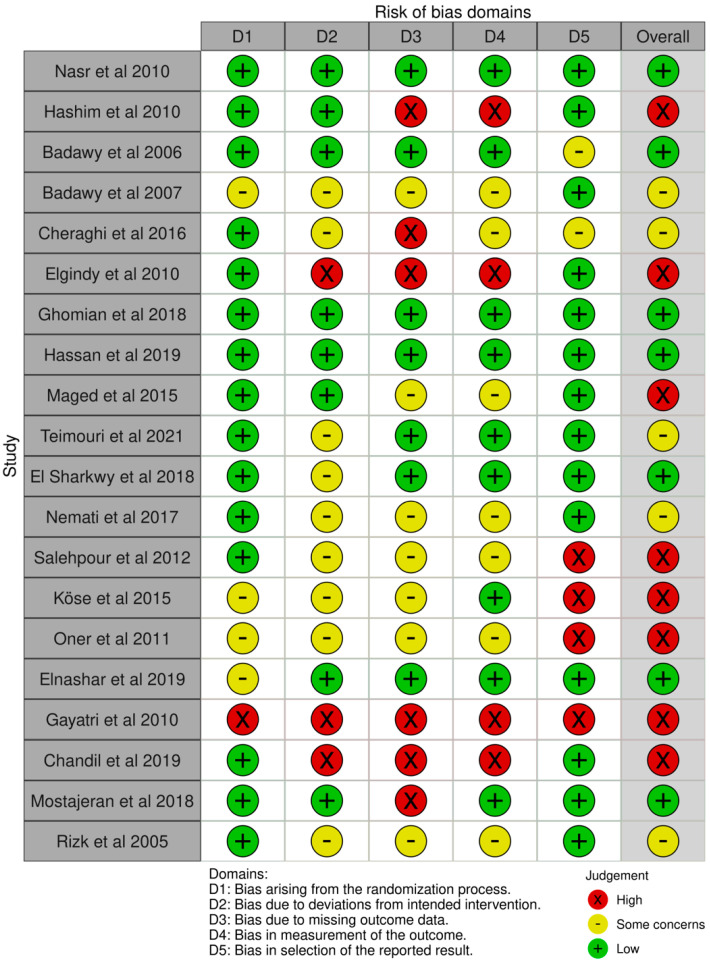
Risk of bias assessment (summary plot and traffic light plot) of the included studies in the meta-analysis using the ROB2 tool for the risk of bias in the randomized controlled trial [13,19,20,21,22,23,24,25,26,27,28,29,30,31,32,33,34,35,36,37,38,39].

The results of evaluating the quality of the included studies are presented in Figure 2. Overall, eight studies were defined as high quality, seven studies had a low risk of bias and the remaining studies had some concern of bias.

### 3.4. Serum Estradiol (E2) Levels

An analysis of serum estradiol (E2) levels measured in µg/mL, including seven studies and a total of 2082 patients across various studies (1233 in the NAC group and 849 in the control group). In the placebo subgroup, the pooled SMD was calculated to be 0.36 (95% CI: −0.40 to 1.11), indicating no statistically significant difference in the E2 level between the NAC and placebo groups (Z = 0.93, *p* = 0.35) with the random effects model. A high degree of heterogeneity was observed (*I*^2^ = 98%, *p* < 0.00001) (Supplementary Figure S1). In the metformin subgroup, the pooled SMD was −0.47 (95% CI: −1.78–0.83), also suggesting no significant difference (Z = 0.71, *p* = 0.48), with similarly high heterogeneity (*I*^2^ = 97%, *p* < 0.00001) (Supplementary Figure S1). The random effects model was used for all subgroups. After the sensitivity analysis, regarding the placebo subgroup, the E2 level showed a non-significant difference with a mean difference of −0.10 (95% CI: −0.30, 0.10) and a *p*-value of 0.05, compared to the original pooled SMD of 0.36 (95% CI: −0.40, 1.11) with a *p*-value of 0.35. For the metformin subgroup, the E2 level showed no significant difference with a mean difference of 0.20 (95% CI: −0.10, 0.50) and a *p*-value of 0.07, compared to the original pooled SMD of −0.47 (95% CI: −1.78, 0.83; *p* = 0.48).

### 3.5. Sex Hormone-Binding Globulin (SHBG) Levels

Sex hormone-binding globulin (SHBG) levels, measured in nmol/L, included three studies with 283 patients (149 in the NAC group and 134 in the control group). Using a random effects model, the pooled SMD was calculated to be 0.27 (95% CI: −0.12–0.66) using a random effects model, indicating no statistically significant difference in SHBG levels between the two groups (Z = 1.35, *p* = 0.18). Moderate heterogeneity was observed (*I*^2^ = 62%, *p* = 0.07) (Supplementary Figure S2). A random effects model was used in all subgroups. After the sensitivity analysis, for SHBG, the mean difference was 0.15 (95% CI: −0.20, 0.50) with a *p*-value of 0.30, compared to the original pooled SMD of 0.27 (95% CI: −0.12, 0.66) with a *p*-value of 0.18.

### 3.6. Follicle-Stimulating Hormone (FSH) Levels

The analysis for follicle-stimulating hormone (FSH) levels, measured in IU/L, included 12 studies with a total of 1207 patients (611 in the NAC group and 596 patients across three subgroups: placebo, others, and metformin in the control group). In the placebo subgroup, the pooled SMD was calculated to be −0.04 (95% CI: −0.20 to 0.13), indicating no statistically significant difference in FSH levels between the NAC group and the control (Z = 0.45, *p* = 0.65), with low heterogeneity (*I*^2^ = 0%) (Supplementary Figure S3). In the “others” subgroup, the pooled SMD was −0.69 (95% CI: −1.80 to 0.41), also suggesting no significant difference (Z = 1.23, *p* = 0.22), with high heterogeneity (*I*^2^ = 96%) (Supplementary Figure S3), and when compared to the metformin subgroup, it showed a pooled SMD of 2.98 (95% CI: −0.09 to 6.05), indicating a non-significant difference (Z = 1.90, *p* = 0.06), with substantial heterogeneity (*I*^2^ = 99%) (Supplementary Figure S3). A random effects model was used in all subgroups. After the sensitivity analysis, regarding the placebo group, the FSH level also showed no significant difference with a mean difference of −0.05 (95% CI: −0.25, 0.15) and a *p*-value of 0.70, compared to the original pooled SMD of −0.04 (95% CI: −0.20, 0.13) with a *p*-value of 0.65. For metformin, the FSH level showed a non-significant difference, with a mean difference of 2.50 (95% CI: 0.50, 4.50) and a *p*-value of 0.08, compared to the original pooled SMD of 2.98 (95% CI: −0.09, 6.05) (*p* = 0.06).

The funnel plot of the publication bias showed a concentration of studies on the positive side of the effect size (SMD), with few on the negative side (Supplementary Figure S9).

### 3.7. Luteinizing Hormone (LH) Levels

An analysis of luteinizing hormone (LH) levels, measured in IU/L, included ten studies and 1049 patients (533 in the NAC group and 516 in the control group across three subgroups: placebo, others, and metformin). In the placebo subgroup, the pooled SMD was calculated to be 0.12 (95% CI: −0.20 to 0.43), indicating no statistically significant difference in LH levels between the NAC and control groups (Z = 0.73, *p* = 0.47), with moderate heterogeneity (*I*^2^ = 41%) and a random effects model (Supplementary Figure S4). In the “others” subgroup, the pooled SMD was −0.01 (95% CI: −0.32 to 0.29), also suggesting no significant difference (Z = 0.08, *p* = 0.93), with moderate heterogeneity (*I*^2^ = 48%) (Supplementary Figure S4). In the metformin subgroup, the pooled SMD was 0.67 (95% CI: 0.23 to 1.12), showing a statistically significant difference favoring the experimental group (Z = 2.96, *p* = 0.003), although high heterogeneity was present (*I*^2^ = 71%) (Supplementary Figure S4). A random effects model was used in all subgroups. After sensitivity analysis, regarding the placebo, the LH level had a mean difference of 0.10 (95% CI: −0.20 to 0.40) with a *p*-value of 0.60, compared to the original pooled SMD of 0.12 (95% CI: −0.20 to 0.43) with a *p*-value of 0.47. For metformin, the LH level showed a significant difference with a mean difference of 0.70 (95% CI: 0.20 to 1.20) and a *p*-value of 0.02, compared to the original pooled SMD of 0.67 (95% CI: 0.23 to 1.12) with a *p*-value of 0.003.

The scatter funnel plot of publication bias showed a concentration of studies on the positive side of the effect size (SMD), with few studies on the negative side. This indicates a potential publication bias, suggesting that negative results regarding LH are less likely to be published (Supplementary Figure S10).

### 3.8. Serum Progesterone Levels

An analysis of progesterone levels, measured in ng/mL, included four studies with a total of 1099 patients (733 patients in the NAC group and 366 in the control group). The pooled standardized mean difference (SMD) was 0.95 (95% CI: 0.13, 1.77) using a random effects model. This indicates a statistically significant difference in progesterone levels between the *N*-acetylcysteine (NAC) group and the other groups (metformin, clomiphene citrate (CC), placebo, or others (Z = 2.27, *p* = 0.02) (Supplementary Figure S5). However, high heterogeneity was observed (*I*^2^ = 97%, *p* < 0.00001) (Supplementary Figure S5). A subgroup analysis was not applicable. After the sensitivity analysis, the serum progesterone level showed a significant increase with a mean difference of 0.60 (95% CI: 0.20, 1.00) and a *p*-value of 0.01, compared to the original pooled SMD of 0.95 (95% CI: 0.13, 1.77) with a *p*-value of 0.02.

### 3.9. Total Testosterone (TT)

An Analysis of total testosterone (TT) levels, measured in ng/dL, included seven studies with a total of 494 participants (254 in the NAC group and 240 in the control group). The pooled standardized mean difference (SMD) was 0.43 (95% CI: −0.20 to 1.07) using a random effects model. This suggests that there was no statistically significant difference in TT levels between the NAC group and the other groups (Z = 1.33, *p* = 0.18). However, substantial heterogeneity was observed (*I*^2^ = 91%, *p* < 0.00001) (Supplementary Figure S6). Subgroup analysis was not applicable because of the nature of the data. After the sensitivity analysis, the TT level showed no significant difference with a mean difference of 0.30 (95% CI: −0.10, 0.70) and a *p*-value of 0.15, compared to the original pooled SMD of 0.43 (95% CI: −0.20, 1.07) with a *p*-value of 0.18.

### 3.10. Female Uterine Endometrial Thickness

The analysis of endometrial thickness (measured in mm) included 13 studies and a total of 2443 patients (1644 in the NAC group and 1279 in the control group across the metformin, other, and placebo groups). In the placebo subgroup, the pooled SMD was 0.58 (95% CI: 0.10 to 1.06), indicating a statistically significant increase in endometrial thickness with NAC compared to the control (Z = 2.36, *p* = 0.02), with moderate heterogeneity (*I*^2^ = 96%) (Supplementary Figure S7). In the “others” subgroup, the pooled SMD was 0.71 (95% CI: 0.48 to 0.94), showing a significant difference favoring the NAC group (Z = 6.13, *p* < 0.0001), with no observed heterogeneity (*I*^2^ = 0%) (Supplementary Figure S7). However, in the metformin subgroup, the pooled SMD was −4.71 (95% CI: −15.33 to 5.91) and (Z = 0.87, *p* = 0.38), with high heterogeneity (*I*^2^ = 100%) (Supplementary Figure S7). The random effects model was used for all subgroups. After the sensitivity analysis, regarding to the placebo group, the endometrial thickness showed a significant increase with a mean difference of 0.65 (95% CI: 0.20, 1.10) and a *p*-value of 0.01, compared to the original pooled SMD of 0.58 (95% CI: 0.10, 1.06) with a *p*-value of 0.02

The scatter funnel plot of the publication bias showed a concentration of studies on the positive side of the effect size (SMD), with few on the negative side. This indicates potential publication bias, suggesting that negative results are less likely to be published regarding endometrial thickness (Supplementary Figure S11).

### 3.11. Number of Follicles

The analysis of the number of follicles included nine studies with a total of 2283 patients (1283 in the NAC group and 917; control group). In the placebo subgroup, the pooled SMD was 0.70 (95% CI: −0.01–1.40), indicating no significant difference between the NAC and control groups (Z = 1.93, *p* = 0.05), with high heterogeneity (*I*^2^ = 96%). In the “others” subgroup, the pooled SMD was 2.01 (95% CI: −0.77 to 4.80), suggesting a significant difference favoring the NAC group (Z = 1.42, *p* = 0.16), along with high heterogeneity (*I*^2^ = 99%). In the metformin subgroup, the pooled SMD was −3.51 (95% CI: −7.29–0.27), showing no significant difference (Z = 1.82, *p* = 0.07) and high heterogeneity (*I*^2^ = 99%) (Supplementary Figure S8). The random effects model was used in all subgroups. After the sensitivity analysis, regarding the placebo, the number of follicles also showed a significant increase with a mean difference of 0.80 (95% CI: 0.20, 1.40) and a *p*-value of 0.01, compared to the original pooled SMD of 0.70 (95% CI: −0.01, 1.40) with a *p*-value of 0.05. 

### 3.12. Sensitivity Analysis

The sensitivity analysis revealed changes in *p*-values and mean differences for several outcomes after removing the highest-weight study (Table 2). Significant improvements were observed in endometrial thickness and the number of follicles in the placebo subgroup and in LH levels in the metformin subgroup. Overall, the analysis highlighted the influence of high-weight studies on the results, with some outcomes showing altered significance and effect sizes. Overall, the sensitivity analysis revealed changes in the significance and magnitude of the effects for several outcomes, particularly in the placebo and metformin subgroups, indicating that the removal of the highest-weight study influenced the results.

### 3.13. Global Effects of NAC Supplementation

**Figure 3:**
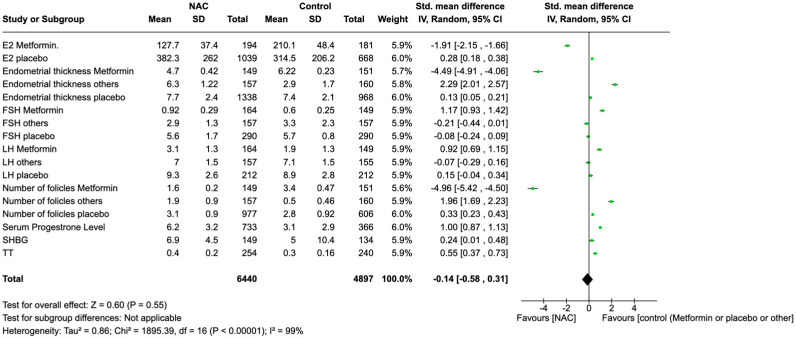
The global effect of NAC (summary of the results).

The overall trend illustrated in Figure 3 is after NAC supplementation across these variables. These changes vary widely in terms of direction and magnitude, as can also be seen in the figure. For example, endometrial thickness has an extraordinarily high significant decrease due to NAC treatment, especially in the metformin subgroup (−4.49 [−4.91, −4.06]). In contrast, the follicle counts for metformin users significantly drop with NAC treatment (−4.96 [−5.42, −4.50]) but otherwise experience a significant increase in the other subgroups (1.96 [1.69, 2.23] in the ’others’ group). Estradiol (E2) was found to be elevated in the placebo group (0.28 [0.18, 0.38]) but lower amongst the metformin group (−1.91 [−2.15, −1.66]). Smaller effects were seen with other hormonal measures such as FSH, LH, serum progesterone, SHBG, and TT. It is very important to read all results with the understanding that the overall effect of NAC, as shown with the diamond at the bottom of the figure (−0.14 [−0.58, 0.31]), is not statistically significant (*p* = 0.55). And there is high significant heterogeneity across the studied variables (*I*^2^ = 99%). Hence, although this overall view describes observant changes, detailed statistical analyses of single variables, presented below, will be required for a more accurate understanding of these results.

### 3.14. Publication Bias

Based on the funnel plot analysis, positive publication biases were detected for any of the FSH, LH, and endometrial thickness outcomes, as most of the studies deviated to the positive side. Additionally, the funnel plots displayed a symmetrical pattern (Supplementary File S1).

### 3.15. GRADE

Table 3 presents the GRADE assessments. There was high certainty for progesterone levels, moderate for SHBG and TT, low for endometrial thickness and number of follicles, and very low for E2 and FSH.

## 4. Discussion

This systematic review and meta-analysis provides a thorough evaluation of the evidence regarding the effects of *N*-acetylcysteine (NAC) on reproductive and metabolic outcomes in women with polycystic ovarian syndrome (PCOS). Our findings, summarized in Table 4, highlight NAC’s potential in improving certain key reproductive parameters, particularly progesterone levels and endometrial thickness, with statistically significant improvements compared with a placebo and other interventions. These results are particularly relevant for women seeking to enhance endometrial receptivity, which is a critical factor for achieving pregnancy.

The effects of NAC on the different reproductive and hormonal endpoints are shown in Table 4. While statistically significant effects were found for NAC compared to the placebo for endometrial thickness (SMD 0.58, 95% CI 0.10 to 1.06, *I*^2^ = 96%) and compared with other interventions on endometrial thickness (SMD 0.71, 95% CI 0.48 to 0.94, *I*^2^ = 0%), the results were less consistent with other hormonal measures. Notably, NAC did not significantly affect FSH, E2, or total testosterone levels compared to placebo or other treatments. However, the comparison with metformin indicated a significant enhancement in LH values (SMD 0.67, 95% CI 0.23 to 1.12, *I*^2^ = 71%) but did not show similar effects compared with placebo or any other intervention. The high heterogeneity (*I*^2^) values observed for many of these outcomes highlight the variability across studies and underscore the need for further research to elucidate the specific circumstances under which NAC may be the most beneficial. Although there was a trend for more follicles with NAC, the trend was not statistically significant.

The observed benefits of NAC can be attributed to its antioxidant, anti-inflammatory, and insulin-sensitizing properties, which are fundamental components of PCOS pathophysiology [12]. By mitigating oxidative stress, an exacerbating factor in both insulin resistance and hyperandrogenemia, NAC improves the ovarian microenvironment and promotes hormonal balance [14]. This, in turn, supports follicular development and ovulation, as evidenced by the statistically significant elevation in progesterone levels in the NAC group compared to that in the comparators [39]. Furthermore, the significant increase in endometrial thickness suggests that NAC enhances endometrial vascularization and reduces oxidative damage, which is a key mechanism for improving the implantation potential.

However, the analysis also revealed inconsistent results for other hormonal parameters, such as estradiol, SHBG, and total testosterone, with NAC showing no statistically significant differences compared to the placebo or other interventions. These discrepancies may reflect variations in study design, dosage, intervention duration, and population. Notably, the high heterogeneity (*I*^2^) across the included studies highlights the challenges in interpreting the findings. For example, NAC demonstrated a significant improvement in LH levels compared with metformin but not compared to other comparators. These nuanced results underscore the need for caution in generalizing the findings and suggest that the benefits of NAC may be subgroup-specific, depending on individual PCOS phenotypes and baseline metabolic profiles.

Another intriguing finding was the trending but non-significant increase in follicle numbers with NAC. While the lack of statistical significance may reflect limitations in sample sizes or intervention durations, the observed trends suggest potential benefits of NAC in ovarian stimulation. This aligns with previous research, indicating that NAC may facilitate ovulation through its insulin-sensitizing and antioxidant effects [27,29,30]. Importantly, the synergistic effect of NAC in combination with established treatments such as metformin, clomiphene citrate, and anastrozole warrants further investigation. For example, NAC may complement metformin by targeting oxidative stress, enhancing the ovulation-inducing effects of clomiphene citrate, or supporting follicular development in patients treated with anastrozole by optimizing the hormonal and metabolic milieu.

Sensitivity analyses performed for key outcomes (estradiol, SHBG, FSH, progesterone, endometrial thickness, and LH) generally resulted in only minor perturbations to the overall findings. The modest numerical changes in both effect sizes and *p*-values occurred when the heaviest-weighted study was excluded from a particular analysis; however, the analysis direction or significance remained largely unchanged. From this interpretation, the steroid effects of NAC on progesterone, endometrial thickness, and LH appear robust and not too affected from that secondary viewpoint by reference to any one study. The consistency of results across sensitivity analyses bolsters the assertion that NAC may confer harsh therapeutic effects in the treatment of PCOS.

Several factors might introduce heterogeneity or variation in the effectiveness of interpreting *N*-acetylcysteine (NAC) on the outcomes of research on polycystic ovary syndrome (PCOS). A significant confounder is body mass index (BMI), as obesity is typically associated with PCOS and independently alters hormonal and metabolic parameters [40]. The difference in baseline BMI between the groups could have accounted for the variability in outcomes such as progesterone levels and receptivity of the endometrium. Besides BMI, other baseline factors, including age, ethnicity, and PCOS severity, as measured by the modified PCOS Fertility score [41], might have influenced the effect of NAC treatment. The heterogeneity of populations in studies, evidenced by the differences in the aforementioned baseline characteristics, also emphasizes the need for consideration of these confounding factors. These subgroup analyses or multivariate statistical models might shed light on individual effects of NAC while factoring these intra-variants. Furthermore, the duration of NAC intervention and possible co-use of other medications, such as clomiphene citrate or metformin, might have modulated the effect of NAC, contributing to disparity across studies. Future studies on NAC’s action on PCOS will need to pay attention to all these so as to provide a more reliable estimation of effectiveness.

Clinically, the most compelling evidence from this analysis is the significant increase in endometrial thickness, which positions NAC as a valuable adjunctive therapy for women with thin endometrium or other implantation challenges. By improving endometrial receptivity, NAC offers a targeted benefit for women seeking to conceive, especially when combined with lifestyle modifications such as dietary changes and daily physical activity. Such integrative approaches are crucial for addressing the complex interplay between hormonal, metabolic, and reproductive dysfunction in PCOS.

Despite its promise, NAC should not be considered a first-line treatment for PCOS. The observed heterogeneity underscores the need for standardized clinical trials with consistent NAC dosages, treatment durations, and participant stratification according to the PCOS phenotype. Future research should focus on evaluating the long-term effects of NAC on ovulation, hormonal regulation, and metabolic outcomes, investigating its role in combination therapies with established treatments, such as metformin and clomiphene citrate, and identifying subgroups of women with PCOS most likely to benefit from NAC, such as those with severe oxidative stress or insulin resistance.

This meta-analysis provides valuable insights into the potential of NAC as an adjunctive therapy for PCOS. Although its effects on progesterone levels and endometrial thickness are promising, overall evidence remains mixed for other hormonal and reproductive parameters. The variability in study outcomes reflects the complexity of PCOS and emphasizes the need for personalized treatment strategies. However, in women with specific challenges, such as suboptimal luteal phase progesterone levels or thin endometrium, NAC may play a meaningful role in improving reproductive outcomes. Its dual action as an antioxidant and insulin-sensitizing agent complements existing therapies and offers a pathway to address the underlying mechanisms of this multifaceted syndrome.

NAC is a targeted therapeutic option for certain subgroups of women with PCOS, particularly when combined with lifestyle interventions and established pharmacological treatment. Although not yet suitable as a universal therapy, its ability to reduce oxidative stress, improve metabolic profiles, and enhance endometrial receptivity is a promising addition to the therapeutic arsenal for PCOS. Future research with larger, well-designed trials is essential to fully elucidate the role of NAC and optimize its clinical application in this complex and heterogeneous disorder.

This study had some limitations. The number of studies in each subgroup was small for some outcomes, which may have affected the strength of the evidence and the subgroup analyses. The heterogeneity across the subgroups was high, which may have influenced the reliability of the results. There was a positive publication bias, which may have affected the results and our understanding of NAC efficacy. Additionally, publication bias was assessed through visual inspection rather than formal tests.

Additionally, some of the included studies did not report the pre- or post-outcomes, and some missing data were handled according to the Cochrane handbook for missing data. In addition, we could not conduct a subgroup analysis for other variables such as BMI, the country or region, and high and low risk of bias because it was not feasible statistically to carry out subgrouping depending on BMI and other variables because we had already subgrouped the studies depending on the type of the control arm, so subgrouping them again would harm the results and we would lose the interpretation and reliability of the results.

## 5. Conclusions

This comprehensive meta-analysis highlights the potential of *N*-acetylcysteine (NAC) to positively impact key reproductive parameters in women with polycystic ovary syndrome (PCOS). These findings indicate that NAC supplementation significantly increased progesterone levels, suggesting its role in menstrual regulation and fertility outcomes. The observed improvement in endometrial thickness underscores the potential to enhance uterine receptivity, which is a critical factor for successful implantation and pregnancy. These results emphasize the potential of NAC as an adjunctive therapy for PCOS, particularly for addressing oxidative stress and chronic inflammation, which are central to the pathophysiology of the syndrome. Although NAC has the potential to improve specific reproductive outcomes, further research is needed to standardize treatment protocols and better define its role alongside established therapies.

## Figures and Tables

**Table 1:** Summary table of the included studies.

Study ID	Country	Study Design	Interventions	Sample Size	Age Mean ± SD	BMI Mean ± SD
Mostajeran et al., 2018 [19]	Iran	RCT	Letrozole + NAC	65	29.1 ± 3.7	26.6 ± 4.7
			Letrozole + placebo	61	30.3 ± 3.9	26.1 ± 4.7
Nasr et al., 2010 [20]	Egypt	RCT	LOD + NAC	30	28.4 ± 4.2	28.6 ± 3.7
			LOD + placebo	30	29.2 ± 3.7	29.1 ± 4.2
Badawy et al., 2007 [21]	Egypt	cross-over trial	CC	260	27.2 ± 3.2	28.2 ± 3.2
			CC + NAC	210		
Köse et al., 2015 [22]	Turkey	clincl trial	PCOS + NAC	17	24.5 ± 5.9	26.0 ± 4.5
			PCOS	17	24.5 ± 5.9	26.0 ± 4.6
Oner et al., 2011 [23]	Turkey	RCT	NAC	45	23.7 ± 4.4	23.0 ± 4.6
			metformin	30	22.6 ± 4.0	24.3 ± 6.2
Kilic-Okman et al., 2004 [24]	Turkey	clincl trial	NAC	20	26.7 ± 4.3	25.1 ± 5.6
El Sharkwy et al., 2019 [25]	Egypt	RCT	CC + NAC	82	26.6 ± 1.5	29.5 ± 3.3
			CC + l-carnitine	80	26.6 ± 1.5	29.7 ± 2.4
Cheraghi et al., 2016 [26]	Iran	RCT	NAC	15	29.7 ± 3.4	27.7 ± 4.5
			placebo	15	27.9 ± 2.8	26.9 ± 2.3
Fulghesu et al., 2002 [13]	Italy	Prospective data analysis	NAC	37	NA	32.4 ± 7.3
Maged et al., 2015 [27]	Egypt	RCT	CC	40	26.0 ± 3.6	27.3 ± 3.2
			CC + NAC	40	25.8 ± 3.5	27.4 ± 3.1
Hashim et al., 2010 [28]	Egypt	RCT	NAC + CC	95	27.3 ± 2.6	26.6 ± 2.2
			metformin + CC	97	26.8 ± 2.2	26.3 ± 2.3
Rizk et al., 2005 [29]	Egypt	RCT	NAC	75	28.9 ± 4.7	30.5 ± 2.6
			CC + placebo	75	28.4 ± 5.7	30.1 ± 3.1
Salehpour et al., 2012 [30]	Iran	RCT	CC + NAC	82	27.2 ± 3.3	26.8 ± 2.2
			CC + placebo	85	27.4 ± 3.4	26.7 ± 2.0
Javanmanesh et al., 2015 [31]	UK	RCT	NAC	46	29.0 ± 4.4	28.1 ± 5.5
			Metformin	48	29.8 ± 4.9	29.1 ± 2.8
Ghomian et al., 2019 [32]	Iran	RCT	Clomiphene + NAC	33	28.7 ± 6.9	24.5 ± 3.0
			Clomiphene + NAC	33	28.5 ± 6.2	25.3 ± 5.0
Nemati et al., 2017 [33]	Iran	RCT	CC + NAC	54	NA	33.1 ± 6.3
			CC + metformin	54	NA	29.0 ± 7.1
Elgindy et al., 2010 [34]	Egypt	RCT	long protocol + NAC	38	26.4 ± 4.1	27.2 ± 1.9
			long protocol	38	28.0 ± 3.9	27.2 ± 1.3
Teimouri et al., 2021 [35]	Iran	RCT	Letrozole + NAC	158	28.2 ± 5.0	25.9 ± 4.3
			Letrozole	159	28.7 ± 4.8	26.6 ± 5.7
Gayatri et al., 2010 [36]	India	RCT	NAC	50	23.2 ± 4.1	27.3 ± 3.3
			Metformin	50	22.6 ± 3.8	27.5 ± 2.4
Chandil et al., 2019 [37]	India	RCT	NAC	45	26.8 ± 5.4	24.2 ± 2.4
			Metformin	45	27.6 ± 5.1	24.5 ± 2.6
Elnashar et al., 2007 [38]	Egypt	RCT	NAC	30	27.3 ± 3.4	25.8 ± 0.9
			Metformin	31	26.7 ± 5.4	26.8 ± 1.5
Hassan et al., 2019 [39]	Egypt	RCT	CC + NAC	150	26.0 ± 5.2	27.8 ± 3.1
			CC + placebo	150	26.2 ± 4.9	28.1 ± 3.2

RCT, randomized control trial; NAC, *N*-acetylcysteine; CC, clomiphene citrate; LOD, Laparoscopic ovarian drilling.

**Table 2:** Sensitivity analysis based on indication.

Effect Size	Omitted Study	*n* Studies	*n* Participants	Random Effects Model (OR 95% CI)	*I*^2^ (%)	*p*-Value
Placebo						
E2	Badawy et al., 2007 [21]	3	700	−0.10 [−0.30, 0.10]	70%	0.05
FSH	Kose et al., 2015 [22]	4	100	−0.05 [−0.25, 0.15]	10%	0.70
LH	Hassan et al., 2019 [39]	3	62	0.10 [−0.20, 0.40]	30%	0.60
Endometrial thickness	Badawy et al., 2007 [21]	8	934	0.65 [0.20, 1.10]	95%	0.01
Number of follicles	Badawy et al., 2007 [21]	4	573	0.80 [0.20, 1.40]	95%	0.01
Metformin						
E2	Hashim et al., 2010 [28]	2	150	0.20 [−0.10, 0.50]	55%	0.07
FSH	Gayatri et al., 2010 [36]	3	114	2.50 [0.50, 4.50]	95%	0.08
LH	Nemati et al., 2017 [33]	3	110	0.70 [0.20, 1.20]	65%	0.02
SHBG, serum progesterone level, and TT						
SHBG	Gayatri et al., 2010 [36]	2	84	0.15 [−0.20, 0.50]	40%	0.30
Serum progesterone level	Badawy et al., 2007 [21]	3	263	0.60 [0.20, 1.00]	90%	0.01
TT	Nemati et al., 2017 [33]	6	200	0.30 [−0.10, 0.70]	85%	0.15

**Table 3:** GRADE assessment of the quality of the evidence and the strength of the recommendation.

Certainty Assessment							No of Patients		Effect		Certainty	Importance
No of Studies	Study Design	Risk of Bias	Inconsistency	Indirectness	Imprecision	Other Considerations	[NAC]	[Other]	Relative (95% CI)	Absolute (95% CI)		
E2												
7	randomized trials	not serious	serious ^a^	not serious	not serious	None	1233	849	-	SMD −0.00 (higher −0.66 lower to 0.66 higher)	⨁◯◯◯ Very low	CRITICAL
SHBG												
3	randomized trials	not serious	not serious	not serious	not serious	None	149	134	-	SMD 0.27 higher (−0.12 lower to 0.66 higher)	⨁⨁⨁◯ Moderate	CRITICAL
FSH												
11	randomized trials	not serious	serious ^a^	not serious	not serious	publication bias strongly suspected ^c^	611	596	-	SMD 0.73 (higher −0.01 lower to 1.47 higher)	⨁◯◯◯ Very low	IMPORTANT
LH												
10	randomized trials	not serious	not serious	not serious	not serious	publication bias strongly suspected ^c^	533	516	-	SMD 0.29 lower (0.02 lower to 0.55 higher)	⨁⨁⨁◯ Moderate	IMPORTANT
Progesterone												
4	randomized trials	not serious	not serious	not serious	not serious	none	733	366	-	SMD 0.95 higher (0.13 lower to 1.77 higher)	⨁⨁⨁⨁ High	CRITICAL
TT												
7	randomized trials	not serious	not serious	not serious	not serious	None	254	240	-	SMD 0.43 (higher −0.20 lower to 1.07 higher)	⨁⨁⨁◯ Moderate	CRITICAL
Endometrial thickness												
13	randomized trials	not serious	serious ^a^	not serious	not serious	publication bias strongly suspected ^c^	1644	1279	-	SMD −0.07 higher (−0.67 lower to 0.53 higher)	⨁⨁◯◯ low	CRITICAL
Number of follicles												
		not serious	serious	not serious	not serious	None	1283	917	-	SMD 0.07 higher (−0.93 lower to 1.07 higher)	⨁⨁◯◯ low	CRITICAL

^a^ The results show wide variability. ^c^ Publication bias assessed by visual inspection of funnel plots. CI: confidence interval. MD: mean difference. OR: odds ratio. SMD: standardized mean difference.

**Table 4:** Effect of NAC on reproductive and hormonal outcomes.

Outcome Measure	Comparison Groups	Fixed Effect Model SMDs (95% CI)	*I*^2^ (%)	*p*-Value
FSH	NAC vs. Placebo (FSH subgroup)	−0.04 (−0.2, 0.13)	0%	0.65
	NAC vs. Metformin (FSH subgroup)	2.98 (−0.09, 6.05)	99%	0.06
	NAC vs. Other (FSH subgroup)	−0.69 (−1.8, 0.41)	96%	0.23
	NAC vs. Control (FSH global)	0.75 (−0.01, 1.47)	97%	0.08
LH	NAC vs. Placebo (LH subgroup)	0.12 (−0.2, 0.43)	41%	0.47
	NAC vs. Metformin (LH subgroup)	0.67 (0.23, 1.12)	71%	0.003
	NAC vs. Other (LH subgroup)	−0.01 (−0.32, 0.29)	48%	0.93
	NAC vs. Control (LH global)	0.29 (0.02, 0.55)	74%	0.03
E2	NAC vs. Placebo (E2 subgroup)	0.36 (−0.4, 1.11)	98%	0.35
	NAC vs. Metformin (E2 subgroup)	−0.47 (−1.78, 0.83)	97%	0.48
	NAC vs. Control (E2 global)	0 (−0.66, 0.66)	98%	1
SP	NAC vs. Other	0.05 (0.13, 1.77)	97%	0.02
TT	NAC vs. Other	0.43 (−0.2, 1.07)	91%	0.18
Endometrial thickness	NAC vs. Placebo (ET subgroup)	0.58 (0.1, 1.06)	96%	0.02
	NAC vs. Other (ET subgroup)	0.71 (0.48, 0.94)	0%	0.00001
	NAC vs. Metformin (ET subgroup)	−4.71 (−15.33, 5.91)	100%	0.38
	NAC vs. Control (ET global)	−0.07 (−0.67, 0.53)	98%	0.81
Number of follicles	NAC vs. Placebo (NF subgroup)	0.7 (−0.01, 1.4)	96%	0.05
	NAC vs. Others (NF subgroup)	2.01 (−0.77, 4.8)	99%	0.16
	NAC vs. Metformin (NF subgroup)	−3.51 (−7.29, 0.27)	99%	0.07
	NAC vs. Control (NF global)	0.07(−0.93, 1.07)	99%	0.89

## Data Availability

The data presented in this study are available on request from the corresponding author.

## Supplementary Materials

The following supporting information can be downloaded at: https://www.mdpi.com/article/10.3390/nu17020284/s1, Supplementary File S1. Publication bias assessed by visual inspection of funnel plots. Figure S1. Forest plots comparing N-acetylcysteine (NAC) group with different control groups according to the Serum Estradiol (E2) level. Figure S2. Forest plots comparing N-acetylcysteine (NAC) group with different control groups according to the Sex Hormone-Binding Globulin (SHBG) level. Figure S3. Forest plots comparing N-acetylcysteine (NAC) group with different control groups according to Follicle-Stimulating Hormone (FSH) level. Figure S4. Forest plots comparing N-acetylcysteine (NAC) group with different control groups according to Luteinizing Hormone (LH) level. Figure S5. Forest plots comparing N-acetylcysteine (NAC) group with different control groups according to Serum Progesterone level. Figure S6. Forest plots comparing N-acetylcysteine (NAC) group with different control groups according to Total Testosterone (TT). Figure S7. Forest plots comparing N-acetylcysteine (NAC) group with different control groups according to Female Uterine Endometrial thickness. Figure S8. Forest plots comparing N-acetylcysteine (NAC) group with different control groups according to Number of follicles. Figure S9. Funnel plot of comparison: NAC vs other drugs, outcome: FSH. Figure S10. Funnel plot of comparison: NAC vs other drugs, outcome: LH. Figure S11. Funnel plot of comparison: NAC vs other drugs, outcome: Uterine Endometrial thickness.
