# Supplementary material for: Efficacy of N-Acetylcysteine in Polycystic Ovary Syndrome: Systematic Review and Meta-Analysis
Source: Nutrients. 2025 Jan 14;17(2):284. doi: 10.3390/nu17020284 (PMC11768055; doi:10.3390/nu17020284)
Supplement: Supplementary file 1 [file nutrients-17-00284-s001.zip › nutrients-3361509-supplementary.pdf]

Study or Subgroup	Experimental Mean	Experimental SD	Experimental Total	Control Mean	Control SD	Control Total	Weight	Std. mean difference IV, Random, 95% CI
**2.1.1 placebo**								
Badawy et al 2006	398	290	404	452.1	290.3	400	14.9%	-0.19 [-0.32 , -0.05]
Badawy et al 2007	452.1	290.3	470	110	30.4	103	14.8%	1.30 [1.07 , 1.52]
Cheraghi et al 2016	69.1	14.6	15	70.2	16.6	15	12.8%	-0.07 [-0.78 , 0.65]
Hassan et al 2019	152.3	122.4	150	112.4	121.8	150	14.8%	0.33 [0.10 , 0.55]
**Subtotal**			**1039**			**668**	**57.2%**	**0.36 [-0.40 , 1.11]**
Test for overall effect: Z = 0.93 (P = 0.35)								
Heterogeneity: Tau² = 0.56; Chi² = 121.53, df = 3 (P < 0.00001); I² = 98%								
**2.1.2 Metformin**								
Hashim et al 2010	256	63.2	95	388	87.3	97	14.5%	-1.72 [-2.05 , -1.39]
Nemati et al 2017	8.05	22.6	54	7.27	4.5	54	14.3%	0.05 [-0.33 , 0.42]
Oner et al 2011	0.4	0.8	45	0.1	1.4	30	14.0%	0.28 [-0.19 , 0.74]
**Subtotal**			**194**			**181**	**42.8%**	**-0.47 [-1.78 , 0.83]**
Test for overall effect: Z = 0.71 (P = 0.48)								
Heterogeneity: Tau² = 1.29; Chi² = 68.38, df = 2 (P < 0.00001); I² = 97%								
**Total**			**1233**			**849**	**100.0%**	**-0.00 [-0.66 , 0.66]**

Test for overall effect: Z = 0.00 (P = 1.00)
Test for subgroup differences: Chi² = 1.16, df = 1 (P = 0.28), I² = 14.2%
Heterogeneity: Tau² = 0.76; Chi² = 243.83, df = 6 (P < 0.00001); I² = 98%

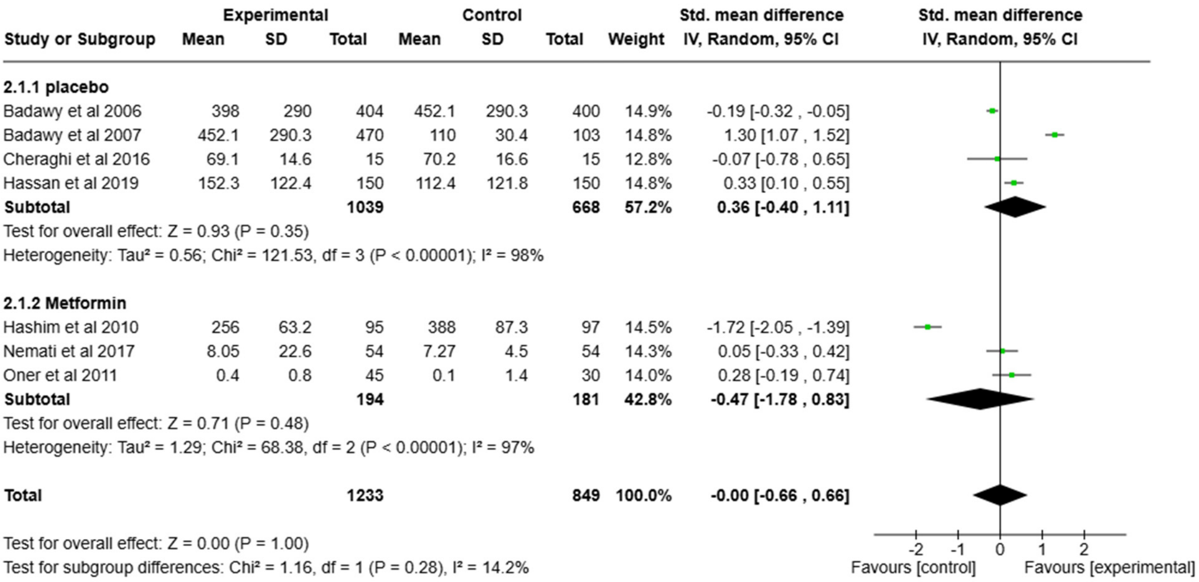

Supplementary Figure S1. Forest plots comparing N-acetylcysteine (NAC) group with different control groups according to the Serum Estradiol (E2) level .

Study or Subgroup	NAC Mean	NAC SD	NAC Total	Others Mean	Others SD	Others Total	Weight	Std. mean difference IV, Random, 95% CI
Gayatri et al 2010	1.04	0.27	50	0.92	0.06	50	34.1%	0.61 [0.21 , 1.01]
Nemati et al 2017	3.1	4.6	54	0.7	12	54	35.5%	0.26 [-0.12 , 0.64]
Oner et al 2011	17.9	9.2	45	19.8	24.8	30	30.5%	-0.11 [-0.57 , 0.35]
**Total**			**149**			**134**	**100.0%**	**0.27 [-0.12 , 0.66]**

Test for overall effect: Z = 1.35 (P = 0.18)
Test for subgroup differences: Not applicable
Heterogeneity: Tau² = 0.07; Chi² = 5.31, df = 2 (P = 0.07); I² = 62%

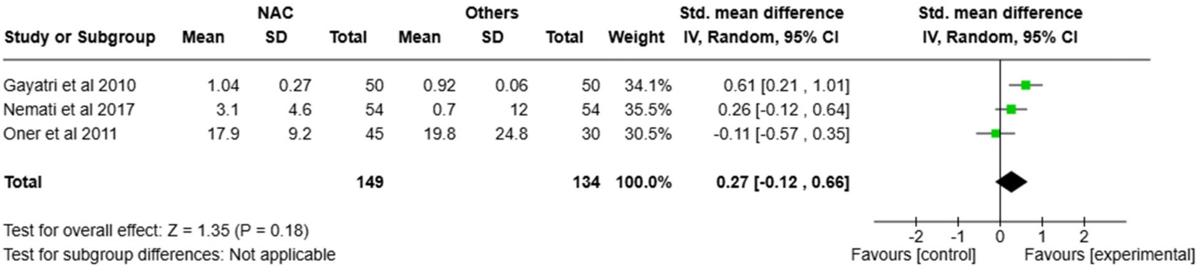

Supplementary Figure S2. Forest plots comparing N-acetylcysteine (NAC) group with different control groups according to the Sex Hormone-Binding Globulin (SHBG) level.

Study or Subgroup	NAC Mean	NAC SD	NAC Total	Others Mean	Others SD	Others Total	Weight	Std. mean difference IV, Random, 95% CI
**1.6.1 Placebo**								
Cheraghi et al 2016	5.3	1.7	15	5.46	0.96	15	8.2%	-0.11 [-0.83 , 0.60]
Elgindy et al 2010	6.12	1.15	38	6.39	1.21	38	8.6%	-0.23 [-0.68 , 0.22]
Hassan et al 2019	6.5	2.2	150	6.6	2.1	150	8.8%	-0.05 [-0.27 , 0.18]
Köse et al 2015	5.07	1.78	17	4.91	1.88	17	8.2%	0.09 [-0.59 , 0.76]
Maged et al 2015	4.46	0.9	40	4.23	1.1	40	8.6%	0.23 [-0.21 , 0.67]
Nasr et al 2010	2.7	1.2	30	2.9	1.7	30	8.5%	-0.13 [-0.64 , 0.37]
**Subtotal**			**290**			**290**	**50.8%**	**-0.04 [-0.20 , 0.13]**
Test for overall effect: Z = 0.45 (P = 0.65)								
Heterogeneity: Tau² = 0.00; Chi² = 2.38, df = 5 (P = 0.80); I² = 0%								
**1.6.2 Others**								
El Sharkwy et al 2019	1.3	0.2	82	1.5	0.1	82	8.7%	-1.26 [-1.59 , -0.92]
Rizk et al 2005	4.7	2.5	75	5.2	4.8	75	8.7%	-0.13 [-0.45 , 0.19]
**Subtotal**			**157**			**157**	**17.4%**	**-0.69 [-1.80 , 0.41]**
Test for overall effect: Z = 1.23 (P = 0.22)								
Heterogeneity: Tau² = 0.61; Chi² = 22.74, df = 1 (P < 0.00001); I² = 96%								
**1.6.3 Metformin**								
Cheraghi et al 2016 (2)	5.3	1.7	15	4.9	1.6	15	8.2%	0.24 [-0.48 , 0.95]
Gayatri et al 2010	0.4	0.02	50	0.1	0.04	50	6.7%	9.41 [8.03 , 10.80]
Nemati et al 2017	0.79	0.3	54	0.07	0.1	54	8.4%	3.20 [2.62 , 3.77]
Oner et al 2011	0.2	0.1	45	0.3	0.2	30	8.5%	-0.67 [-1.14 , -0.19]
**Subtotal**			**164**			**149**	**31.8%**	**2.98 [-0.09 , 6.05]**
Test for overall effect: Z = 1.90 (P = 0.06)								
Heterogeneity: Tau² = 9.63; Chi² = 247.27, df = 3 (P < 0.00001); I² = 99%								
**Total**			**611**			**596**	**100.0%**	**0.73 [-0.01 , 1.47]**

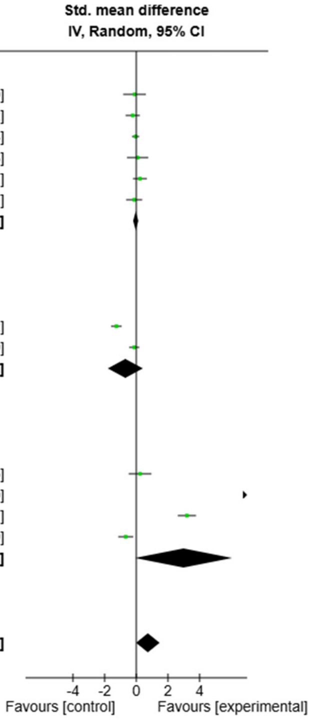

Test for overall effect: Z = 1.92 (P = 0.05)
Test for subgroup differences: Chi² = 5.05, df = 2 (P = 0.08), I² = 60.4%
Heterogeneity: Tau² = 1.62; Chi² = 360.51, df = 11 (P < 0.00001); I² = 97%

Supplementary Figure S3. Forest plots comparing N-acetylcysteine (NAC) group with different control groups according to Follicle-Stimulating Hormone (FSH) level.

Study or Subgroup	NAC Mean	NAC SD	NAC Total	Others Mean	Others SD	Others Total	Weight	Std. mean difference IV, Random, 95% CI
**1.5.1 Placebo**								
Cheraghi et al 2016	11.51	2.47	15	10.77	2.41	15	7.0%	0.30 [-0.43 , 1.02]
Hassan et al 2019	7.3	2.1	150	6.6	2.1	150	13.2%	0.33 [0.10 , 0.56]
Köse et al 2015	9.88	3.88	17	11.23	4.39	17	7.4%	-0.32 [-1.00 , 0.36]
Nasr et al 2010	17.7	4.6	30	18.3	5.5	30	9.5%	-0.12 [-0.62 , 0.39]
**Subtotal**			**212**			**212**	**37.1%**	**0.12 [-0.20 , 0.43]**
Test for overall effect: Z = 0.73 (P = 0.47)								
Heterogeneity: Tau² = 0.04; Chi² = 5.10, df = 3 (P = 0.16); I² = 41%								
**1.5.2 Others**								
El Sharkwy et al 2019	3.8	0.8	82	3.7	0.6	80	12.2%	0.14 [-0.17 , 0.45]
Rizk et al 2005	10.4	2.2	75	10.8	2.4	75	12.0%	-0.17 [-0.49 , 0.15]
**Subtotal**			**157**			**155**	**24.2%**	**-0.01 [-0.32 , 0.29]**
Test for overall effect: Z = 0.08 (P = 0.93)								
Heterogeneity: Tau² = 0.02; Chi² = 1.91, df = 1 (P = 0.17); I² = 48%								
**1.5.3 Metformin**								
Cheraghi et al 2016 (2)	11.51	2.47	15	10.68	1.88	15	7.0%	0.37 [-0.35 , 1.09]
Gayatri et al 2010	2.3	0.1	50	1.01	1.63	50	10.6%	1.11 [0.69 , 1.53]
Nemati et al 2017	0.66	0.7	54	0.45	0.92	54	11.2%	0.26 [-0.12 , 0.63]
Oner et al 2011	4	2.8	45	1.9	1.2	30	9.8%	0.90 [0.42 , 1.39]
**Subtotal**			**164**			**149**	**38.6%**	**0.67 [0.23 , 1.12]**
Test for overall effect: Z = 2.96 (P = 0.003)								
Heterogeneity: Tau² = 0.14; Chi² = 10.31, df = 3 (P = 0.02); I² = 71%								
**Total**			**533**			**516**	**100.0%**	**0.29 [0.02 , 0.55]**

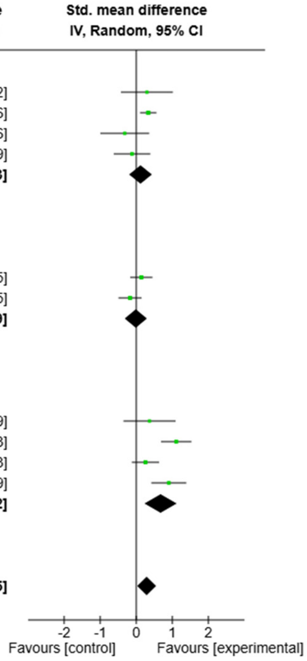

Test for overall effect: Z = 2.14 (P = 0.03)
Test for subgroup differences: Chi² = 6.37, df = 2 (P = 0.04), I² = 68.6%
Heterogeneity: Tau² = 0.12; Chi² = 35.20, df = 9 (P < 0.0001); I² = 74%

Supplementary Figure S4. Forest plots comparing N-acetylcysteine (NAC) group with different control groups according to Luteinizing Hormone (LH) level.

Study or Subgroup	NAC Mean	NAC SD	NAC Total	Others Mean	Others SD	Others Total	Weight	Std. mean difference IV, Random, 95% CI
Badawy et al 2007	6.4	2.3	470	2.3	1.2	103	25.5%	1.91 [1.67 , 2.15]
Elgindy et al 2010	1.09	0.69	38	0.91	0.65	38	24.1%	0.27 [-0.19 , 0.72]
Hassan et al 2019	6.7	5.2	150	4.6	5.1	150	25.5%	0.41 [0.18 , 0.64]
Rizk et al 2005	6.87	5.6	75	1.8	2.2	75	24.9%	1.19 [0.84 , 1.53]
**Total**			**733**			**366**	**100.0%**	**0.95 [0.13 , 1.77]**

Test for overall effect: Z = 2.27 (P = 0.02)
Test for subgroup differences: Not applicable
Heterogeneity: Tau² = 0.67; Chi² = 91.59, df = 3 (P < 0.00001); I² = 97%

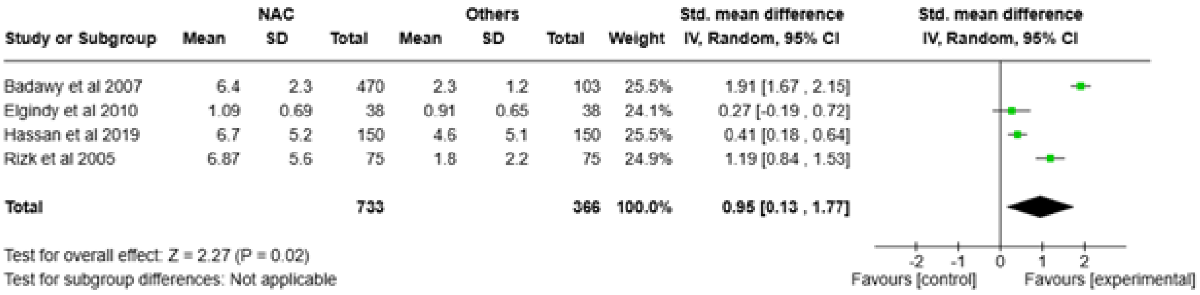

Supplementary Figure S5. Forest plots comparing N-acetylcysteine (NAC) group with different control groups according to Serum Progesterone level.

Study or Subgroup	NAC Mean	NAC SD	NAC Total	Others Mean	Others SD	Others Total	Weight	Std. mean difference IV, Random, 95% CI
Chandil et al 2019	0.38	0.14	45	0.17	0.05	45	14.5%	1.98 [1.47 , 2.49]
Cheraghi et al 2016	1.11	0.54	15	1.1	0.5	15	13.3%	0.02 [-0.70 , 0.73]
Cheraghi et al 2016 (2)	1.11	0.54	15	1.1	0.5	15	13.3%	0.02 [-0.70 , 0.73]
Elnashar et al 2007	0.2	0.3	30	0.36	0.1	31	14.4%	-0.71 [-1.23 , -0.19]
Gayatri et al 2010	0.5	0.03	50	0.3	0.29	50	14.9%	0.96 [0.55 , 1.38]
Nemati et al 2017	0.1	0.1	54	0.05	0.03	54	15.0%	0.67 [0.28 , 1.06]
Oner et al 2011	0.2	0.1	45	0.2	0.1	30	14.7%	0.00 [-0.46 , 0.46]
**Total**			**254**			**240**	**100.0%**	**0.43 [-0.20 , 1.07]**

Test for overall effect: Z = 1.33 (P = 0.18)
Test for subgroup differences: Not applicable
Heterogeneity: Tau² = 0.66; Chi² = 66.97, df = 6 (P < 0.00001); I² = 91%

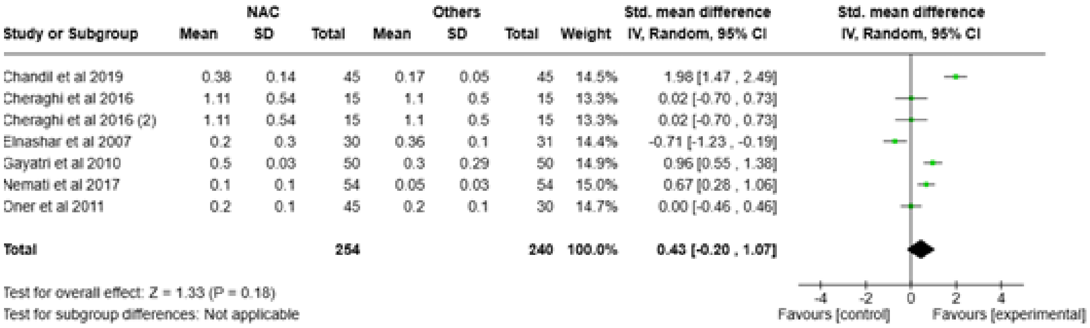

Supplementary Figure S6. Forest plots comparing N-acetylcysteine (NAC) group with different control groups according to Total Testosterone (TT).

Study or Subgroup	NAC Mean	NAC SD	NAC Total	Others Mean	Others SD	Others Total	Weight	Std. mean difference IV, Random, 95% CI
**1.2.1 Placebo**								
Badawy et al 2006	8.3	2.2	404	8.8	3.1	400	8.1%	-0.19 [-0.32 , -0.05]
Badawy et al 2007	7.8	3.1	470	4.3	1.2	103	8.0%	1.22 [1.00 , 1.45]
Cheraghi et al 2016	9.4	0.9	15	8.9	1.6	15	7.2%	0.37 [-0.35 , 1.10]
Elgindy et al 2010	8.92	1.17	38	8.71	1.08	38	7.7%	0.18 [-0.27 , 0.64]
Ghomian et al 2019	7.47	1.6	33	7.58	2.1	33	7.7%	-0.06 [-0.54 , 0.42]
Hassan et al 2019	5.7	1.4	150	5.6	1.2	150	8.0%	0.08 [-0.15 , 0.30]
Maged et al 2015	7.3	1.1	40	5.4	0.6	40	7.6%	2.12 [1.57 , 2.68]
Nasr et al 2010	8.7	1.3	30	6.2	1.4	30	7.5%	1.83 [1.22 , 2.44]
Teimouri et al 2021	7.6	2.5	158	7.9	2.7	159	8.0%	-0.12 [-0.34 , 0.11]
**Subtotal**			**1338**			**968**	**69.9%**	**0.58 [0.10 , 1.06]**
Test for overall effect: Z = 2.36 (P = 0.02)								
Heterogeneity: Tau² = 0.49; Chi² = 193.78, df = 8 (P < 0.00001); I² = 96%								
**1.2.2 Others**								
Rizk et al 2005	5.9	0.7	75	4.9	1.9	75	7.9%	0.69 [0.37 , 1.02]
Salehpour et al 2012	6.6	1.69	82	5.4	1.61	85	7.9%	0.72 [0.41 , 1.04]
**Subtotal**			**157**			**160**	**15.8%**	**0.71 [0.48 , 0.94]**
Test for overall effect: Z = 6.13 (P < 0.00001)								
Heterogeneity: Tau² = 0.00; Chi² = 0.02, df = 1 (P = 0.90); I² = 0%								
**1.2.3 Metformin**								
Hashim et al 2010	6.8	0.2	95	9.4	0.3	97	6.5%	-10.14 [-11.20 , -9.07]
Nemati et al 2017	0.9	0.8	54	0.5	0.1	54	7.8%	0.70 [0.31 , 1.09]
**Subtotal**			**149**			**151**	**14.3%**	**-4.71 [-15.33 , 5.91]**
Test for overall effect: Z = 0.87 (P = 0.38)								
Heterogeneity: Tau² = 58.52; Chi² = 352.02, df = 1 (P < 0.00001); I² = 100%								
**Total**			**1644**			**1279**	**100.0%**	**-0.07 [-0.67 , 0.53]**
Test for overall effect: Z = 0.24 (P = 0.81)								
Test for subgroup differences: Chi² = 1.23, df = 2 (P = 0.54), I² = 0%								
Heterogeneity: Tau² = 1.15; Chi² = 582.22, df = 12 (P < 0.00001); I² = 98%								

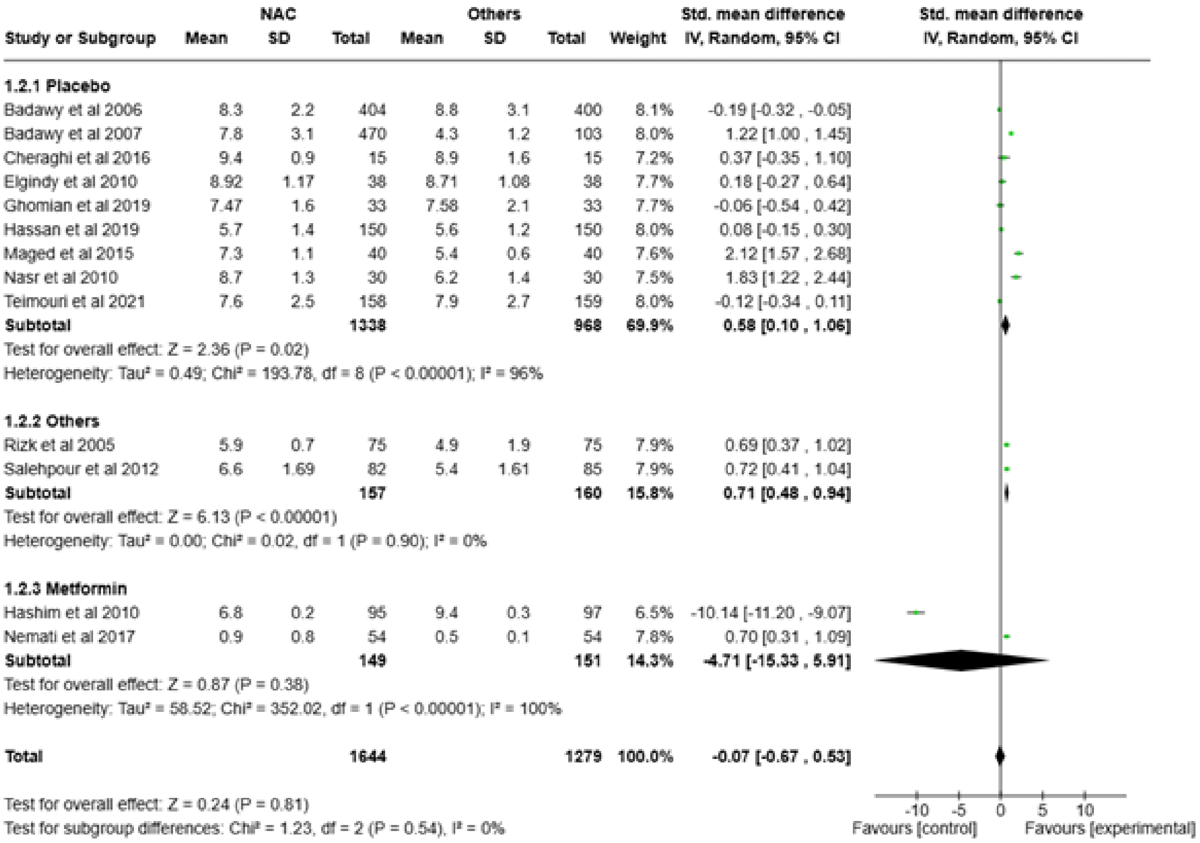

Supplementary Figure S7. Forest plots comparing N-acetylcysteine (NAC) group with different control groups according to Female Uterine Endometrial thickness.

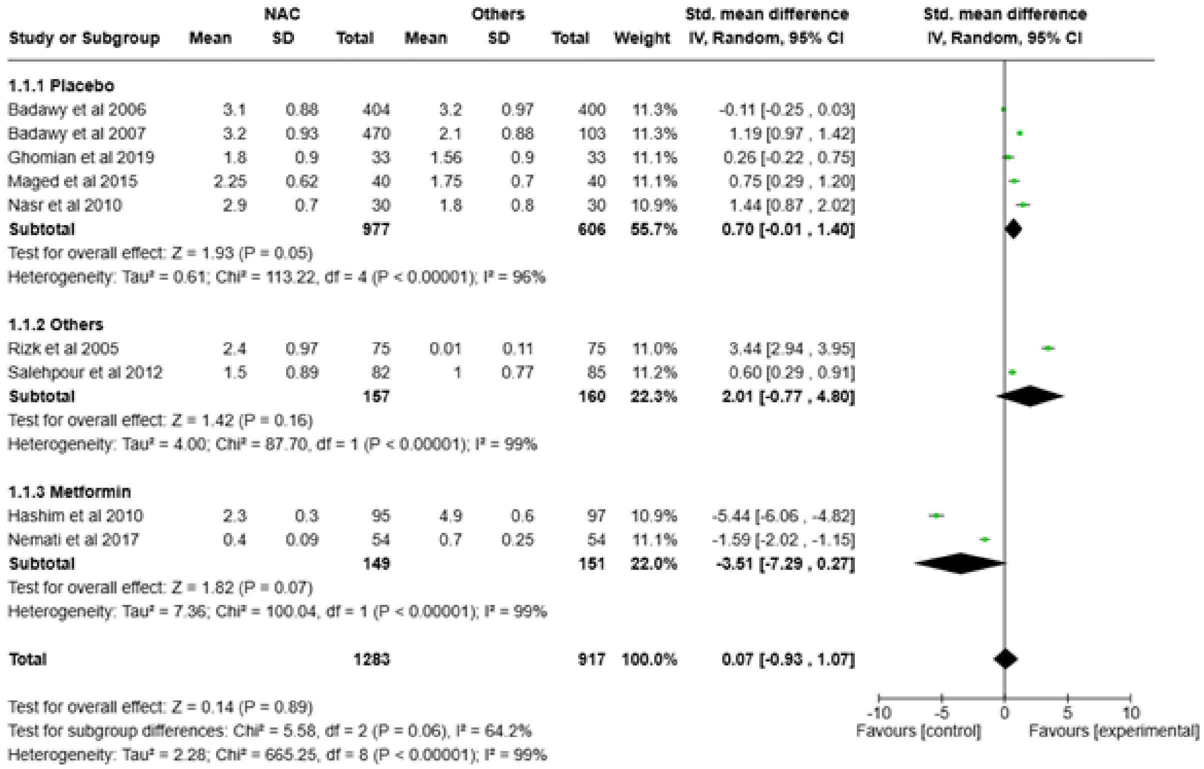

Study or Subgroup	NAC Mean	NAC SD	NAC Total	Others Mean	Others SD	Others Total	Weight	Std. mean difference IV, Random, 95% CI
**1.1.1 Placebo**								
Badawy et al 2006	3.1	0.88	404	3.2	0.97	400	11.3%	-0.11 [-0.25 , 0.03]
Badawy et al 2007	3.2	0.93	470	2.1	0.88	103	11.3%	1.19 [0.97 , 1.42]
Ghomian et al 2019	1.8	0.9	33	1.56	0.9	33	11.1%	0.26 [-0.22 , 0.75]
Maged et al 2015	2.25	0.62	40	1.75	0.7	40	11.1%	0.75 [0.29 , 1.20]
Nasr et al 2010	2.9	0.7	30	1.8	0.8	30	10.9%	1.44 [0.87 , 2.02]
**Subtotal**			**977**			**606**	**55.7%**	**0.70 [-0.01 , 1.40]**
Test for overall effect: Z = 1.93 (P = 0.05)								
Heterogeneity: Tau² = 0.61; Chi² = 113.22, df = 4 (P < 0.00001); I² = 96%								
**1.1.2 Others**								
Rizk et al 2005	2.4	0.97	75	0.01	0.11	75	11.0%	3.44 [2.94 , 3.95]
Salehpour et al 2012	1.5	0.89	82	1	0.77	85	11.2%	0.60 [0.29 , 0.91]
**Subtotal**			**157**			**160**	**22.3%**	**2.01 [-0.77 , 4.80]**
Test for overall effect: Z = 1.42 (P = 0.16)								
Heterogeneity: Tau² = 4.00; Chi² = 87.70, df = 1 (P < 0.00001); I² = 99%								
**1.1.3 Metformin**								
Hashim et al 2010	2.3	0.3	95	4.9	0.6	97	10.9%	-5.44 [-6.06 , -4.82]
Nemati et al 2017	0.4	0.09	54	0.7	0.25	54	11.1%	-1.59 [-2.02 , -1.15]
**Subtotal**			**149**			**151**	**22.0%**	**-3.51 [-7.29 , 0.27]**
Test for overall effect: Z = 1.82 (P = 0.07)								
Heterogeneity: Tau² = 7.36; Chi² = 100.04, df = 1 (P < 0.00001); I² = 99%								
**Total**			**1283**			**917**	**100.0%**	**0.07 [-0.93 , 1.07]**
Test for overall effect: Z = 0.14 (P = 0.89)								
Test for subgroup differences: Chi² = 5.58, df = 2 (P = 0.06), I² = 64.2%								
Heterogeneity: Tau² = 2.28; Chi² = 665.25, df = 8 (P < 0.00001); I² = 99%								

Supplementary Figure S8. Forest plots comparing N-acetylcysteine (NAC) group with different control groups according to Number of follicles.

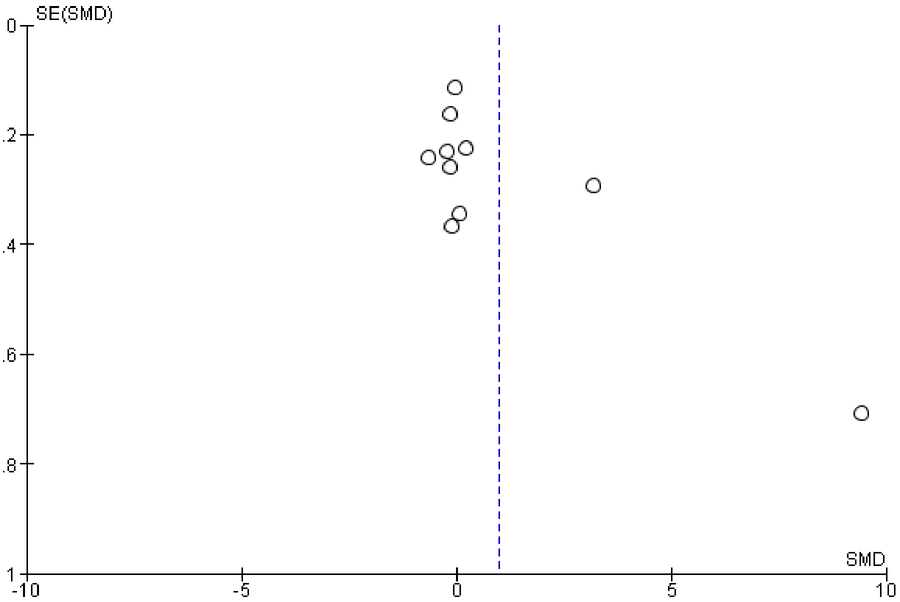

Supplementary Figure S9. Funnel plot of comparison: NAC vs other drugs, outcome: FSH.

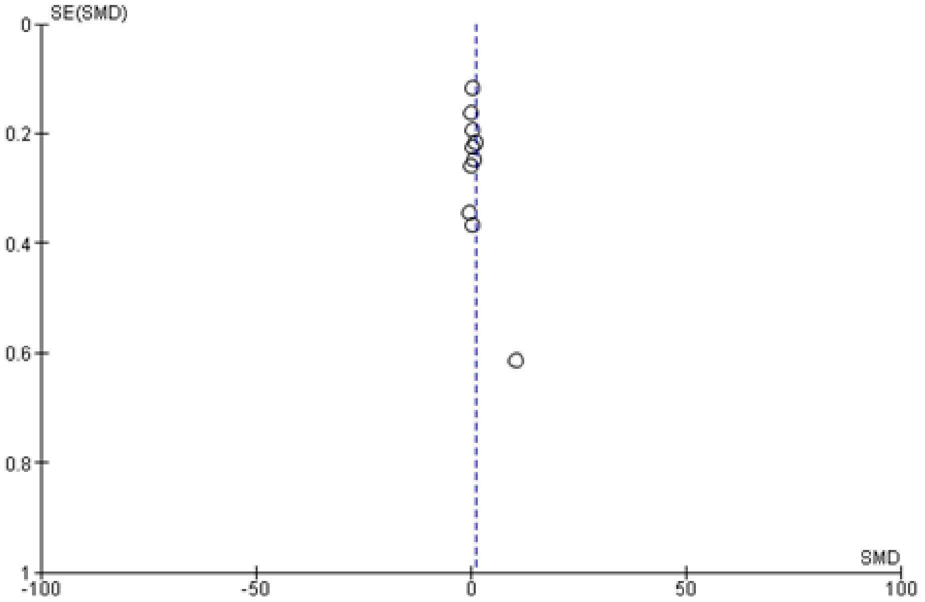

Supplementary Figure S10. Funnel plot of comparison: NAC vs other drugs, outcome: LH

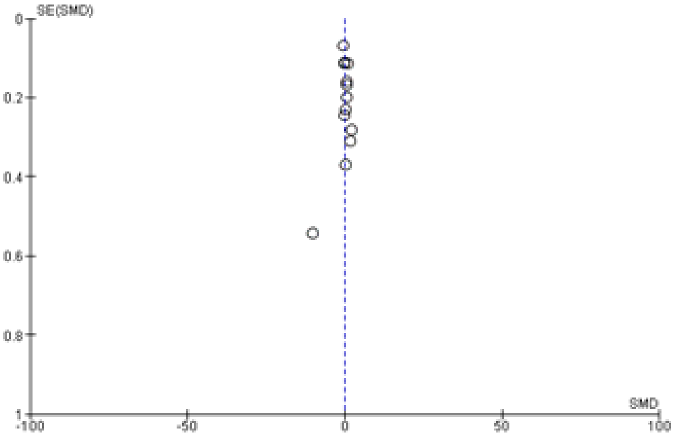

Supplementary Figure S11. Funnel plot of comparison: NAC vs other drugs, outcome: Uterine Endometrial thickness.
